# Mindfulness for psychosis groups; description and preliminary evaluation of a novel routine care pathway in Hong Kong

**DOI:** 10.1186/s13033-020-00415-1

**Published:** 2020-11-10

**Authors:** K. T. Ting, Wendy Tam, Pamela Jacobsen

**Affiliations:** 1 Department of Psychology, University of Bath, Bath, BA2 7AY UK; 2 Department of Clinical Psychology, Kowloon Hospital, Kowloon, Hong Kong

**Keywords:** Psychotic disorders, Mindfulness, Cognitive behaviour therapy, Outpatients, Hong Kong

## Abstract

**Background:**

There is no current guidance on where Mindfulness for Psychosis groups should best be situated within care pathways. The objectives of this paper are to (1) describe a novel care pathway tested out in a psychiatric outpatient service in Hong Kong, and (2) to present feasibility outcomes on attendance and drop-out, and routine clinical outcomes.

**Methods:**

A new mindfulness pathway was set up, for service users with psychosis who had first completed a course of Cognitive Behavioural Therapy for psychosis (CBTp). After attending an orientation ‘taster’ session, service users could then attended a 4-session weekly Mindfulness for Psychosis group, followed by optional monthly follow-up sessions.

**Results:**

A high proportion of service users referred into the pathway (19/22; 86%) went on to attend a Mindfulness for Psychosis group after attending an orientation ‘taster’ session. Attendance at group sessions was high, with all participants attending at least 2/4 group sessions, and no drop-outs. Attendance at monthly follow-up groups was also high, with 84% (16/19) attending at least one monthly follow-up. Routine clinical outcome data showed a reduction in negative symptoms of psychosis, and an increase in mindfulness and mindful responding in daily life, from pre- to post group.

**Conclusions:**

Offering service users with psychosis the opportunity to attend a mindfulness for psychosis group after completing a course of CBTp was highly acceptable, as evidenced by high attendance, and low drop-out. Possible benefits in terms of improving negative symptoms may be particularly important in promoting recovery through improved everyday functioning.

## Introduction

In the acute phase of a psychotic disorder, people may struggle with distressing symptoms, such as voices, and delusional beliefs [1]. However, even after an acute mental health crisis has resolved, it is very common for people to experience persistent residual, or attenuated, psychotic symptoms [2, 3]. People may also experience barriers to re-integrating into society due to the stigma of their diagnosis [4], and the on-going impact of symptoms on everyday functioning [5].

Mindfulness-based therapies offer an alternative way for people to relate to chronic psychotic symptoms, with the aim of reducing distress and impairment to functioning, rather than elimination of symptoms per se [6]. Through mindfulness practice, participants learnt to relate differently to their internal experiences, including phenomena such as voices, or paranoid thoughts, by bringing the attention to the here-and-now with a non-judgmental, compassionate attitude. People are invited to just observe the transient nature of experience with openness and a welcoming attitude, even towards experiences which may be perceived as distressing or unwanted [7]. Qualitative studies of Mindfulness for Psychosis groups [8–11] show that participants commonly report feeling more able to observe and let go of their habitual reaction towards difficult experiences. Participants in these studies also report that through this process they achieved greater self-acceptance, gaining a greater sense of self-identity outside of just ‘psychosis’. Participants reported finding the group process itself therapeutic, as they felt supported by others in the group who had similar experiences to them.

Mindfulness for Psychosis groups have been evaluated in a variety of different health-care settings internationally, including the United Kingdom (UK) [12], Spain [13], Hong Kong [14], and China [15]. There have been several systematic reviews and meta-analyses published in the field since 2013 [16–19]. The most recent meta-analyses have reported large effect sizes for overall symptomatology in favour of mindfulness (9 RCTs, SMD = 0.8; [16]), and significant overall benefit specifically for psychotic symptoms, with a small to medium effect size (8 RCTs, SMD = 0.29; [18]).

Despite these encouraging findings, it is not yet known where mindfulness-based therapies are best situated in the care pathway which is a challenge to further implementation. There is a need to share information on how Mindfulness for Psychosis is currently being implemented internationally, so that feasible care pathways can be tested out in different healthcare systems, and subject to further evaluation. The focus of this paper is therefore on describing a novel service configuration that was pioneered in a psychiatric outpatient service in Hong Kong. A care pathway was developed which involved offering people group-based Mindfulness for Psychosis, *after* they had already completed a course of CBTp. Offering the mindfulness group at this point in the care pathway has several advantages. Firstly, people have the opportunity of learning mindfulness skills, complementary to those skills already learnt through cognitive therapy, which could help them cope with on-going distressing psychotic symptoms. Secondly, group-based therapy offers additional benefits which are particularly important to promote recovery and social reintegration, such as feeling accepted and understood by others [20]. Thirdly, attendees are offered the opportunity to attend monthly follow-up group sessions after the end of their group, thereby improving continuity of care, which is highly-valued by users of psychiatric services [21, 22].

The aims of this paper are therefore to (1) describe the care pathway of Mindfulness for Psychosis in a routine psychiatric service in Hong Kong, (2) to present feasibility outcomes on attendance and drop-out, and preliminary data on routine clinical outcomes.

## Method

### Description of service

The care pathway was developed in a psychiatric outpatient service in a public hospital in Hong Kong. Local citizens could access the public psychiatric service, which is government subsidized, with a referral from a general practitioner (GP). All service users in the psychiatric service were under the care of a psychiatrist, who initiate referrals for psychological intervention, as an adjunct to pharmacotherapy. After an initial screening session, service users who were assessed as being suitable for a psychological therapy were offered a course of individual CBTp as a first-line psychological treatment. As part of the new care pathway, after service users had completed their course of CBTp, they were subsequently given the opportunity to attend an orientation ‘taster’ session for the mindfulness group. Service users were then given the option of enrolling in a 4-week Mindfulness for Psychosis group after the orientation session if they felt it would be helpful for them. This was offered in addition to their usual care, which continued as normal, including any prescribed medication and regular review with their psychiatrist and care team.

### Participants

In this paper, we describe the care pathway and routine outcomes for service users who attended 1 of 6 mindfulness groups, which were run between June 2017 and October 2019 (28 months). All group attendees completed pre- and post-group measures as part of standard service evaluation.

### Overview of the mindfulness for psychosis group

The Mindfulness for Psychosis group was aimed at improving well-being and promoting recovery, in the presence of any on-going psychotic symptoms, rather than aiming to eliminate or suppress such symptoms. The group was run in line with the adaptations suggested by Chadwick [7] to make the practices safe and appropriate for people with psychosis, including using shorter practices (max 10 min) with more frequent verbal guidance. This makes the practices of manageable length, and provides more frequent ‘anchoring’ to the here-and-now which helps prevent people getting lost in their experiences. Participation in all in-session practices was invitational, and participants were given explicit permission to take a break, or stop participating at any time if they felt they needed to. The group included both mindfulness of breathing, and mindfulness of movement (including mindful walking). Other brief practices, such as mindful eating were also incorporated from the standard Mindfulness-Based Cognitive Therapy curriculum [23]. The group had 4 weekly sessions (in addition to the orientation session), and each session lasted for 1 h 15 min. To ensure the course was delivered with the required competence and fidelity to the required adaptions to make the course safe for people with psychosis, the group facilitators were a Clinical Psychologist with expertise in psychosis and over 5 years of experience delivering mindfulness-based interventions, alongside a psychology assistant with personal experience in mindful practice. Table 1 summarises the curriculum for the orientation session, weekly groups, and monthly follow-ups.

**Table 1:** Group sessions curriculum

	Session content
Orientation Session	Introduction Brief practice of mindful seeing, hearing, touching and smelling Brief practice of mindful breathing Research findings and feedback from previous participants Q&A
Four weekly sessions	
1	Check in Ground rules Introduction and expectations for session Raisin exercise and enquiry Mindful breathing and enquiry Set home practice + handout Mindful eating everyday Mindful consumption of food Mindful breathing
2	Check in Ground rules Mindful breathing and enquiry Home practice review Mindful movement and enquiry Set home practice + handout Mindful movement Mindfulness of one activity Mindful breathing everyday
3	Check in Ground rules Mindful breathing and enquiry Homework review Mindful walking and enquiry Set home practice + handout Mindful walking Mindful breathing
4	Check-in Ground rules Mindful breathing Home practice review Sharing and closing
Monthly follow-up meetings	
	Check in Ground rules Mindful breathing/movement/walking Practice review (in-session and home practice) Mindful living Closing

On completion of the 4-week group, participants were invited to attend monthly follow-up meetings, along with other graduates from previous groups. The monthly meetings kept the same familiar structure as the four weekly sessions, led by the same teachers. A mindful living practice was introduced in the monthly follow-ups, which focused on informal mindfulness practices such as practicing mindfulness when doing household chores, appreciating nature etc. The aim of this was to support the application and cultivation of mindfulness skills in daily living, by encouraging both ongoing formal, and informal, practices.

### Routine outcome measures

Participants completed outcome measures at Week 1 (pre-group) and Week 4 (post-group). Clinical measures included negative symptoms, depressive symptoms, mindfulness, and general well-being. We included 2 different measures of mindfulness to assess any changes in both dispositional mindfulness (C-SMQ), and a contextual measure of mindful responding in daily life (DMRS). In addition, in-session measures (stress bubbles) were completed at the beginning and end of each monthly follow-up session. Participants who had completed a group were also invited to a focus group to give some qualitative feedback on their experiences. One focus group with 4 participants was held in April 2019, facilitated by a psychology assistant, who also conducted individual phone interviews with a further 2 participants.

1. *Brief Negative Symptoms Scale* (BNNS; [24]). The BNSS is a 13-item, clinician-rated scale of negative symptoms of psychosis (e.g. blunted affect, anhedonia), which is assessed via a brief semi-structured interview. Each item is rated on a scale from 0 (absent)—6 (severe), with total scores ranging from 0–78, with higher scores indicating a greater severity of symptoms. The scale has good inter-rater and test–retest reliability, good internal consistency, and established construct validity [24].
2. *Beck Depression Inventory-II—Chinese version* (C-BDI-II; [25]). The BDI-II is a 21-item, self-report measure for assessing depressive symptoms [26]. Each item is scored on a scale from 0–3, with total scores ranging from 0 to 63, with higher scores indicating more severe symptoms. The Chinese translation has been validated in a Hong Kong community sample and displays good psychometric properties, including high internal consistency (α = 0.94) [27].
3. *Southampton Mindfulness Questionnaire—Chinese version* (C-SMQ; [28]). The SMQ is a 16-item, self-report questionnaire which measures the degree to which individuals mindfully respond to distressing thoughts and images. It has been validated for use in in a clinical sample of people experiencing distressing psychotic symptoms [29]. Each item is rated on a scale from 0 (totally disagree) to 6 (totally agree), with total scores ranging from 0–96, with higher scores indicated a greater degree of mindful responding. As well as a total score, four sub-scales can be calculated (mindful observation, letting go, absence of aversion, non-judgement). The Chinese translation has excellent test–retest reliability and good internal consistency [28].
4. *Daily Mindful Responding Scale* (DMRS; [30]) (2015). The DMRS is a 4-item self-report questionnaire for measuring mindfulness in a daily life. Each item is rated on a scale from 1 (rarely) to 10 (often), with scores averaged across all 4 items so that total scores range from 1–10, with higher scores indicated a greater degree of everyday mindfulness. It has been shown to be a reliable and valid measure of everyday mindfulness, and is sensitive to change over time for people undergoing a mindfulness-based intervention [30]. As no published Chinese translation was available, the measure was translated with permission from the authors for use in this study.
5. *Short Warwick-Edinburgh Mental Well-being Scale* (C-SWEMWBS; [31]). The SWEMWBS is a 7-item self-report questionnaire measuring well-being [32]. Each item is rated on a scale from 1 (none of the time) to 5 (all of the time), with total scores ranging from 7 to 35, with higher scores indicating higher levels of wellbeing. The Chinese translation has high levels of internal consistency, good test–retest reliability, and good construct validity [31].
6. *Stress Bubbles*. For those attending monthly follow-up meetings, participants completed ‘stress bubbles’ as brief in-session measures, at the beginning and end of each session. This is a self-report, visual analogue scale with ‘bubbles’ of increasing sizes from 1 (not at all) to 6 (extremely). There were separate scales for ‘stress’ and ‘distress’ arising from unwanted thoughts/images/voices, with higher score reflecting higher level of stress and distress. This scale has been shown to be sensitive to within-session change for Mindfulness for Psychosis groups, in both inpatient [33] and community settings [34].

### Data analysis

Quantitative outcome data were analysed using SPSS Version 23, using paired t tests to compare pre- and post-scores on outcome measures. Alpha was set at p = 0.05.

## Results

### Attendance and drop-out

Twenty-two service users were referred into the Mindfulness for Psychosis care pathway and attended a mindfulness orientation session from June 2017 to October 2019. All service users had already completed a course of CBTp (either individual or group), except for one participant who was referred for relapse prevention only. Nineteen participants then decided to go on and take part in the 4-week mindfulness group (19/22; 86%). Six groups were run from 2017 to 2019, with between 3 and 4 participants in each group. Attendance at the group was very high, with a mean average attendance of 3.79 sessions (range 2–4 sessions per participant), with 16/19 (84%) participants attending all 4 sessions. No participants ever walked out of a session, or left early, during the four weekly sessions indicating high acceptability of the mindfulness practices. No participants dropped out of a group once they started attending.

Sixteen out of these 19 participants (84%) attended at least one monthly follow-up session after completion of the 4-session program (15 monthly follow-ups were conducted in total). Six participants attended regularly (≥ 75% attendance), 3 participants attended occasionally (< 75% attendance), and the remaining 7 participants either attended once or dropped out later due to work commitments, hospitalization, or participation in other community services. The average group size of monthly follow-up sessions was 5 participants (range 3–7).

### Description of participants who attended a 4-week group (N = 19)

All 19 participants had a diagnosis of a schizophrenia-spectrum disorder (ICD-10 code: F20-F29) and had an average length of contact with mental health services of 17 years (range 2–37). There were 6 male (32%) and 13 female (68%) participants, with an average age of 49 years (range 25–67), all of whom were of Chinese ethnicity. Ten participants were married (47%), and 9 were single (53%). Nine participants had completed secondary-school education (47%), 2 had diploma-level qualifications (11%), 4 were university graduates (21%), and 4 participants had unknown educational status (21%). Nine participants were employed (47%), 5 were retired (26%), 3 were homemakers (16%), 1 was a student (5%), and 1 was unemployed (5%).

### Routine clinical outcomes

Paired outcome data were available for 18/19 (95%) participants (1 participant did not complete post-group measures due to absence at session 4). Paired sample *t *tests were conducted, to compare pre- and post- scores (Table 2). On symptom measures, there was evidence of a reduction in mean scores of negative symptoms of psychosis from pre-post group (BNSS; pre = 9.17, post = 4.17, p = 0.016). There was a small increase in mean mindfulness score as assessed by the C-SMQ from pre-post group (pre = 46.61, post = 49.83, t(17) = − 2.77, p = 0.013), and a small increase in mindful responding in daily life (pre = 5.29, post = 6.50, t(17) = − 2.17, p = 0.045). There was no change in general well-being, or mean depression score, from pre-post group (C-SWEMWBS; pre = 21.33, post = 22.05; C-BDI-II; pre = 17.72, post = 16.56).

**Table 2:** Mean scores on clinical outcome measures (pre-post group, N = 18)

Outcome measures	Pre-group score (N = 18)	Post-group score (N = 18)	Paired sample T test
(1) Negative symptoms (BNSS; range 0–78)			
Mean (SD)	9.17 (12.55)	4.17 (7.07)	t(17) = 2.68, p = 0.016*
(2) Depressive symptoms (C-BDI-II; range 0–63)			
Mean (SD)	17.72 (10.54)	16.56 (10.79)	t(17) = 0.702, p = 0.492
(3) Mindfulness of thoughts and images (C-SMQ; range 0–96)			
Total score			
Mean (SD)	46.61 (7.44)	49.83 (6.90)	t(17) = − 2.77, p = 0.013*
Sub-scales (range 0–24)			
Mindful observation			
Mean (SD)	14.06 (3.33)	14.17 (3.54)	t(17) = − 0.14, p = 0.890
Letting go			
Mean (SD)	11.17 (3.55)	12.00 (3.53)	t(17) = − 1.55, p = 0.140
Absence of aversion			
Mean (SD)	12.33 (3.48)	13.17 (3.26)	t(17) = − 1.61, p = 0.127
Non-judgement			
Mean (SD)	9.06 (2.90)	10.5 (3.17)	t(17) = − 2.28, p = 0.036*
(4) Daily mindfulness (DMRS; range 1–10)			
Mean (SD)	5.29 (1.92)	6.50 (1.42)	t(17) = − 2.17, p = 0.045*
(5) Well-being (C-SWEMWBS; range 7–35)			
Mean (SD)	21.33 (5.16)	22.06 (5.24)	t(17) = − 0.959, p = 0.351

*p < 0.05

Over the course of the 15 monthly follow-up meetings, 53 paired sets of in-session stress bubble ratings were collected in total from 16 participants (with some participants contributing over multiple sessions), with no indication of an increase in mean average stress or distress ratings from pre-post session (pre-stress = 3.07, post-stress = 2.79; pre-distress = 2.96, post-distress = 2.73).

### Focus group/phone interviews: qualitative outcomes

Participants reported finding mindfulness useful for regulating their emotions. For example, one participant said:—“Mindfulness helps to soothe my emotion and take care of my mental illness”. Another participant commented that after taking part in the mindfulness group, they felt they had become less irritable and had fewer outbursts of temper. Participants also reported change in their general attitude to life. One participant commented:—“Mindfulness helps me to slow down in daily life. It reminds me to keep calm and stay relaxed”. Another participant shared that they felt they had learnt to ‘cherish’ everything in life. Most participants shared that they would like to continue mindful practice after the 4-week group. For those who attended the follow-up group, participants spoke about finding support from other group members, especially when sharing difficulties encountered in keeping up a regular practice. Coming to follow-up meetings was also seen as a helpful reminder of that they had learnt during the mindfulness group. No participants reported any unwanted, or adverse effects from taking part in the mindfulness group.

## Discussion

This study describes the development and evaluation of a new care pathway for Mindfulness for Psychosis in an outpatient psychiatric service in Hong Kong. Offering service users with psychosis the opportunity to attend a Mindfulness for Psychosis group after completing a course of CBT was highly acceptable, as evidenced by high attendance, and low drop-out rates. Attendance at monthly follow-up sessions was also high, indicating the potential value of offering people the opportunity for on-going support in maintaining and deepening their mindfulness practice. Qualitative feedback from service users highlighted what people perceived the subjective benefits of the mindfulness group to be, including helping with emotional wellbeing in everyday life. People also valued the monthly follow-up sessions, to help them remember to keep practicing mindfulness, and to get support from others in the group.

These findings are consistent with previous small-scale service evaluation projects, which have shown that Mindfulness for Psychosis groups are highly acceptable to service users in routine practice, with low drop-out rates in both community [34, 35] and inpatient settings [33, 36]. Mindfulness interventions are increasingly popular with the general public to improve general wellbeing, as well as being applied in health services. Therefore, offering mindfulness groups may be viewed by psychiatric service users as a relatively low-stigma intervention which anyone could benefit from, regardless of diagnosis status. This is important as many service users may not necessarily view their difficulties as a mental health illness, but nonetheless may be interested in psychological interventions to reduce distress [37]. In the current study, participants’ subjective reports on why they kept attending the group, and perceived benefits, are also consistent with previous findings from qualitative studies which highlight the importance of learning new ways to relate to difficulties, and getting support from others with similar experiences in the group [8–11]. The value of offering on-going support in the form of monthly follow-up groups also fits with the concept of ‘recovery’ as an ongoing process, rather than a clear endpoint which is achieved after a particular therapy or intervention [38]. Service users in this study were clearly highly motivated to attend sessions, including optional follow-up sessions, as previous studies have shown much higher rates of drop-out from group therapies of around 20% [39]. There may be several reasons for the low drop-out rate in the current study, including the ‘opt-in’ nature of the way the mindfulness group was offered, and the opportunity to try an orientation ‘taster’ session first. The importance of encouraging service user choice and a feeling of autonomy has been previously highlighted as a facilitating factor which promotes regular group attendance in a qualitative study of 67 people who had attended therapy groups in the community [40].

The routine clinical outcome measures should be interpreted with appropriate caution, given the small sample size. The lack of a control group also means any changes over time could have arisen from other factors such as spontaneous recovery over time, rather than the mindfulness group itself. However, it is interesting to note that the pattern of change was not the same across all measures. Most importantly, there was no indication of deterioration on any of the measures, which fits with previous data from meta-analyses of randomized controlled-trials that there is no evidence of serious harm or adverse effects arising from Mindfulness for Psychosis [16, 18]. There was a small positive improvement both on a measure of mindfulness of distressing thoughts and images (C-SMQ) and mindful responding in daily life (DMRS). There was also evidence of an improvement in negative symptoms from pre-post group. This may be an important benefit of the group warranting further research, given the association between negative symptoms and poorer long-term functioning in psychosis [41]. Interestingly, findings from the 2 latest meta-analyses of Mindfulness for psychosis RCTs present equivocal findings on negative symptoms, with Jansen et al. [16] reporting evidence of a beneficial treatment effect based on 3 RCTs (SMD = 0.41, p < 0.001), whilst Louise et al. [18] report no overall effect based on 4 RCTs (SMD = 0.09, p = 0.561). These meta-analyses only share 1 study in common for negative symptoms however [13], due to different inclusion criteria for each review, which likely explains the difference in findings given the studies included are largely non-overlapping. In the current study, there was no evidence of any improvement in depressive symptoms from pre-post group. Again, previous findings are mixed, with Louise et al. [18] reporting evidence of a positive benefit on depressive symptoms (3 RCTs, SMD = 0.39, p = 0.011), but Jansen et al. [16] reporting a contradictory finding, but based on only 1 RCT [12] (SMD = 0.38, p = 0.07).

As discussed above, as this was a service evaluation project designed only to evaluate feasibility of the pathway, the findings should be interpreted within the limitations of this approach. Larger studies, using a randomized controlled design with an appropriate control group, would be required in future research to establish clinical efficacy of the pathway. Furthermore, the findings of the feasibility of the care pathway in Hong Kong may not be generalizable to other countries, due to substantial differences in how health care services are structured and run in different countries. How a Mindfulness for Psychosis group is best situated within a larger care pathway will also depend on what other treatments and therapies are available. Taking the UK as an example, although CBT for psychosis is recommended as part of standard care, overall implementation rates remain low [42, 43]. Therefore, if Mindfulness for Psychosis is only offered *after* CBT for psychosis, implementation will be limited if availability of CBT is limited. On the issue of how to evaluate routine services, there are always limitations to the size of the battery of outcome measures which can be feasibly implemented. However, we acknowledge that we did not collect data on several key outcomes for psychosis, such as positive symptoms, self-rated recovery, or quality of life. Service user input will be valuable in helping clinicians to prioritise the most important measures to include to evaluate routine mindfulness services in the future. In the Hong Kong service, psychiatrists acted as the gate-keepers to the mindfulness group as they made the referrals; further research is therefore needed to assess wider acceptability of a mindfulness group, given that we do not know what proportion of the total eligible service user group might have attended given the opportunity to be referred.

In conclusion, it was possible to set up a new care pathway for Mindfulness for Psychosis within a routine Psychiatric outpatient service in Hong Kong, which has now run successfully for over 2 years. Data on attendance and drop-out indicate high acceptability of the pathway for the first 22 service users to enter the pathway. Routine outcome measures indicate possible benefits in terms of reducing negative symptoms and increasing mindfulness and mindful responding in daily life. Care pathways in different countries will need to be set up according to local need and available resources; given these factors, the pathway described in this paper should not therefore be necessarily viewed as a one-size-fits-all approach to service planning and delivery.

## Data Availability

The datasets used and/or analysed during the current study are available from the corresponding author on reasonable request.

## Abbreviations

CBTp: Cognitive-behavioural therapy for psychosis
TAU: Treatment as usual
UK: United Kingdom
US: United States
